# Control flow graph based code optimization using graph neural networks

**DOI:** 10.3389/frobt.2026.1731740

**Published:** 2026-03-11

**Authors:** Melih Peker, Ozcan Ozturk

**Affiliations:** 1 Computer Engineering Department, Bilkent University, Ankara, Türkiye; 2 Faculty of Engineering and Natural Sciences, Sabanci University, Istanbul, Türkiye

**Keywords:** code optimization, compilers, FLAG, GCC, graph neural networks

## Abstract

Selecting a good set of optimization flags requires extensive effort and expert input. While most of the prior research considers using static, spatial, or dynamic features, some of the latest research directly applied deep neural networks to source code. We combined the static features, spatial features, and deep neural networks by representing source code as graphs and trained Graph Neural Network for automatically finding suitable optimization flags. We created a dataset of 12000 graphs using 256 optimization flag combinations on 47 benchmarks. We trained and tested our model using these benchmarks, and our results show that we can achieve a maximum of 48.6% speed-up compared to the case where all optimization flags are enabled.

## 1 Introduction

Compiler optimizations allow programmers to create more efficient object codes by applying various transformations to the source code at compile time. Those optimizations have four main goals: reducing execution time, minimizing compile time, consuming fewer resources, and minimizing the code size (G. Team, 2025). Such optimizations generally minimize resource usage so that the resulting programs run faster. To maximize efficiency, most compilers have lots of optimization options. For instance, the GCC compiler has over 200 predefined optimization options (G. Team, 2025), and LLVM has 150 (LLVM, 2024). Choosing the best optimizations for a program is very important for utilizing the hardware, minimizing the cost of resources, and creating scalable deployments. Since there are many optimization options and it is essential to choose the best ones for efficiency, selecting the proper optimizations for a particular program is very complex.

**FIGURE 1:**
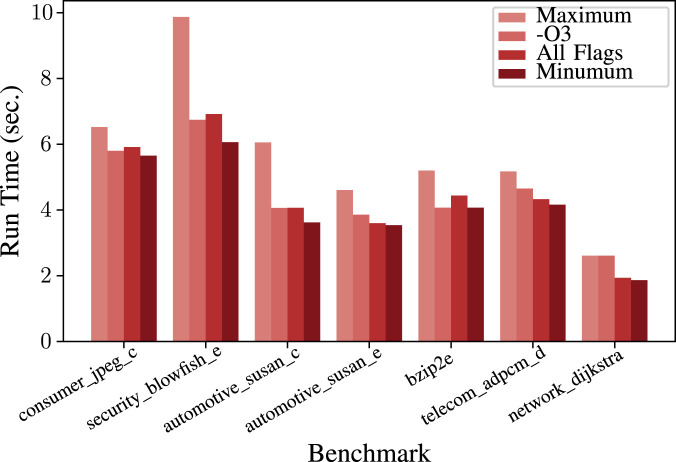
Run time of some benchmarks with −O3 , with all optimization flags enabled along with maximum and minimum.

To make it easier, GCC offers six predefined optimization levels; −O1 , −O2 , −O3 , −Os , −Ofast , and −Og (G. Team, 2025). These levels selectively choose and utilize some of the compiler optimization options. Although it is easier to choose between these levels rather than 200 flags, it is still hard to choose the best one. Moreover, using all optimizations or choosing the most aggressive level (for GCC, −O3 ) does not guarantee the best performance (Branco and Henriques, 2015). For instance, when we compare the execution time of benchmarks using all the flag combinations and −O3 baseline, we observe that neither −O3 nor enabling all the optimization flags gives the best possible run time. Therefore, as shown in Figure 1, using predefined optimizations usually is not good enough to bring the best achievable application-specific performance (Fursin et al., 2011).

Since it is hard to know whether a program will benefit from an optimization flag or a combination of optimization flags, choosing the best set of flags requires expert knowledge and many attempts. Because more than 200 optimization flags exist, there are more than $2^{200}$ possible combinations, and it is nearly impossible to find the best one by trying all of them.

For a long time, researchers have tried to find a fast, robust, and easy method for selecting the best optimization flags. The traditional way of choosing the best optimizations includes finding hot spots in code by testing it and analyzing the assembly code (Plotnikov et al., 2013). This approach requires expert knowledge and time. For automatic tuning of the compiler optimizations, some researchers proposed using *genetic algorithms* to generate random sets and then find the best group by mutating those sets according to a cost function (Plotnikov et al., 2013; Pérez Cáceres et al., 2017). Some researchers proposed employing *iterative search* algorithms to narrow down the search space and then construct the best optimizations from that space (Sher et al., 2014; Ogilvie et al., 2017; Blackmore et al., 2017). Although those methods provide promising results, like achieving better run time performance than −O3 (Blackmore et al., 2017), they are not fast enough. Finding the best optimization from those large sets still requires countless executions for finding the best one.

In recent years, learning-based models have been used for modeling complex systems and understanding complex concepts like languages and images (Young et al., 2018). Since machine learning (ML) models are proven to solve complex problems, ML-based approaches are also used to understand source codes (Fursin et al., 2011; Malik, 2010; Pekhimenko and Brown, 2011). Although extracting features and learning patterns from source codes is not that straightforward, many research efforts employ machine learning for automatic code optimization tasks (Fursin et al., 2011; Malik, 2010). One of the famous ones, Milepost GCC, used machine learning to find the best optimizations by manually extracting features and learning patterns from those features (Fursin et al., 2011). Different research groups focused on spatial information of the source code and extracted spatial features from data flow graphs (DFG) and control flow graphs (CFG) for representing source codes as a fixed feature vector (Malik, 2010; Park et al., 2012). While most of these researches used manual feature extraction, some researchers find similarities between language and source codes and use natural language processing (NLP) models for representing source code as tokenized feature vectors (Peng et al., 2015; Mendis et al., 2019). Representing source codes as fixed-size vectors is a very sophisticated task, and the authors aimed to tackle these challenges.

GNN-based methods have been widely used for program analysis and optimization. Prior work models programs using representations such as ASTs or intermediate representations to learn semantic embeddings. These approaches support tasks including variable and method name prediction, data type inference, performance prediction, and compiler optimizations like device mapping and thread coarsening. In the literature, GNNs are used to enable learning language-independent program representations that generalize across multiple downstream optimization tasks. In contrast, our approach applies GNNs to identify optimal compiler optimization flags using features derived from the control-flow graph. Unlike prior work, we jointly model basic blocks as node features and control-flow relationships as graph edges during GNN training. This explicit combination of node-level static properties and edge-level structural information enables the model to capture richer program semantics and make more informed optimization decisions.

This paper combines the benefits of hand-crafted features, spatial information, and automatically extracted features. We specifically utilize Graph Neural Networks (GNNs) on program CFGs to extract features and predict the best compiler flags. Our specific contributions can be listed as follows:

- We extract meaningful hand-crafted features from the source code to represent the program as a fixed-size feature vector. We dive deep into the basic block level to extract the most meaningful information and create feature vectors for each basic block.
- We utilize the spatial information for preserving and using CFG features such as loops, jumps, calls, and other characteristics.
- We generate graphs by combining hand-crafted features with spatial information for representing the source code as directed graphs with node features.
- We create a GNN model and train the model using the graphs that we generated. Using our generated graphs, the GNN model suggests the best compiler flags.
- Models have been tested in different domains and with other benchmarks to show the applicability in different scenarios. Our approach is not discipline-specific and can be applied to various cases. For instance, the GNN model trained using a math benchmark (Pouchet, 2012) can be used on a benchmark with much more complex programs (Guthaus et al., 2001).

The rest of the paper is organized as follows. The next chapter discusses the necessary background. Chapter IV gives the high level view, whereas Chapter V explains the details of our GNN-based approach. Chapter VI discusses the experimental setup and results, and Chapter VII concludes the paper.

## 2 Program analysis

**FIGURE 2:**
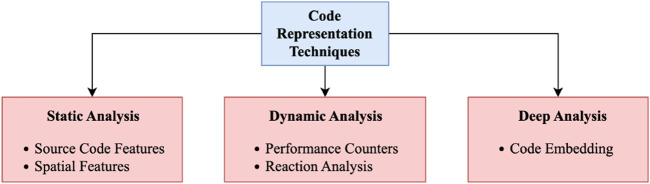
Code representation techniques used in literature.

Learning-based models learn patterns between input and output from training data and try to find similar patterns in test data to predict the outcome (Young et al., 2018). These approaches usually incur expensive one-time training but are inexpensive when applied to new programs. Therefore, machine learning models need a good source code representation to find similarities between them. Symbolic encoding should be used in the applications to see the similarities between code snippets (Peng et al., 2015; Mendis et al., 2019). Most ML models need fixed-size feature vectors as input for finding the similarities between programs using linear and non-linear combinations between those features. Therefore, researchers mainly focus on finding a good source code representation. Research efforts in this domain can be classified into three main categories, as seen in Figure 2.

### 2.1 Static analysis

Static analysis extracts features directly from the source code or the compiler intermediate representation (IR). These features are extracted **without** running the code itself, which is why they are called static features. Some examples of these features are the number of branches, number of loops, variable types, number of arithmetic operations, and other static properties.

#### 2.1.1 IR features

Most of the static feature extraction process uses the compiler intermediate representation (IR) to avoid using irrelevant information such as dead code. IR is an important target for optimization since it contains useful structural and execution information about the application.

#### 2.1.2 Spatial features

One year after the Milepost, Malik showed that spatial-based features are better for characterizing the programs instead of using only the source code features (Malik, 2010). These features show how different instructions are distributed within a program, which is crucial for instruction scheduling and register allocation. More specifically, the author uses DFGs, where each node represents an instruction, and each edge shows the data dependency between two instructions. This way, the spatial information of the program can be extracted and used in training.

**FIGURE 3:**
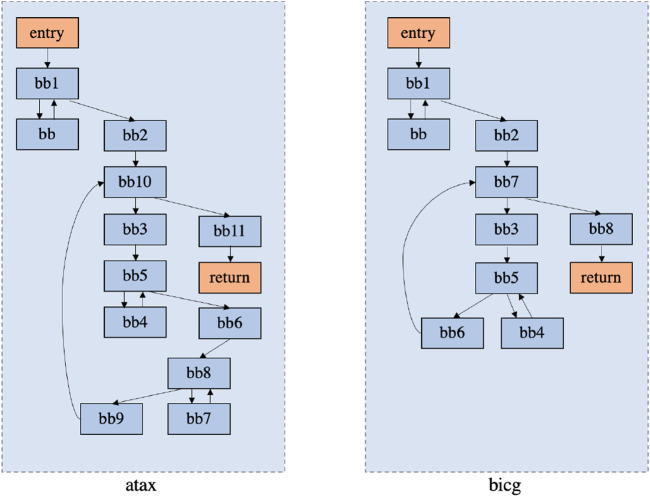
Similarities between the CFG of two different algorithms from the Polybench benchmark suite (Abella-González et al., 2021).

Thanks to the research that demonstrated the benefits of using dependence graphs in characterizing loops, they are also used as features for representing source codes. For instance, Figure 3 shows an example of the similarity between the CFG graphs of similar algorithms. Similar programs may have similar CFG structures, so the CFG information can be used as an indicator when characterizing the programs.

### 2.2 Dynamic analysis

Static code features may contain information about the code segments that are rarely executed, which can confuse the machine learning model (Wang and O’Boyle, 2018). Some program information, such as the loop bounds, depends on the program input, which can only be obtained during the execution time. Also, static code features may not precisely capture the application behavior in the run time environment (such as resource contention and I/O behavior) (Cavazos et al., 2007). This highly depends on the computing environment, such as the number of available processors and co-running workloads (Wang and O’Boyle, 2018).

To address such run time changes, dynamic analysis is usually preferred. The run time information contains behaviors on specific hardware components with particular inputs, hot code, loop counts, etc. Although it is more complex than collecting static features, the dynamic analysis may represent the source code better in specific environments and execution scenarios (Cavazos et al., 2007).

### 2.3 Deep analysis

Manual feature extraction methods require expert input, which is even more complex at the source code level. Specifically, static features are not always enough to represent a program, and extracting dynamic features is exhaustive since it requires run time information (Peng et al., 2015). Moreover, finding relations between hand-crafted features is a complex procedure. Therefore, recent studies focused on automatic feature extraction methods using deep neural networks.

### 2.4 Program features for automatic code optimization

Commonly used parts for automatic code optimization can be classified into four main categories as shown in Table 1. Tree-graph-based features give insight into the execution order of the program. These features show the loop structures, branching nodes, etc., which offers a great idea about the program structure and data flows. This category is divided into two subsections since while some work uses CFGs for tree-graph-based features, some use DFGs for extracting spatial information. Dynamic features can be used if the task optimizes code for specific hardware. Reaction analysis, loop counts, and cache hit/miss information are valuable for particular hardware but not helpful for different architectures. Finally, deep analysis methods use abstract syntax trees or word embedding to analyze the source code. Those methods do not use hand-crafted features. Instead, they extract their own features.

**TABLE 1:** Common features used in machine learning techniques for automatic code optimization.

Commonly Used Features	Source Code Features	Loop Information	(Fursin et al., 2011) (Pekhimenko and Brown, 2011) (Namolaru et al., 2010) (Blackmore et al., 2015) (Koseki et al., 1997) (Cavazos et al., 2007)
		Basic Block Information	(Fursin et al., 2011) (Sher et al., 2014) (Pekhimenko and Brown, 2011) (Park et al., 2012) (Namolaru et al., 2010) (Blackmore et al., 2015)
		# of Basic Blocks	(Fursin et al., 2011) (Sher et al., 2014) (Pekhimenko and Brown, 2011) (Park et al., 2012) (Namolaru et al., 2010) (Blackmore et al., 2015)
		# of Floating Point Operations	(Fursin et al., 2011) (Sher et al., 2014) (Pekhimenko and Brown, 2011) (Kulkarni and Cavazos, 2012) (Namolaru et al., 2010) (Blackmore et al., 2015)
		# of Memory Operations	(Fursin et al., 2011) (Namolaru et al., 2010) (Blackmore et al., 2015) (Ashouri et al., 2016)
		# of Integer Instructions	(Fursin et al., 2011) (Sher et al., 2014) (Pekhimenko and Brown, 2011) (Kulkarni and Cavazos, 2012) (Namolaru et al., 2010) (Blackmore et al., 2015)
		# of Array Instructions	(Fursin et al., 2011) (Pekhimenko and Brown, 2011) (Kulkarni and Cavazos, 2012) (Namolaru et al., 2010) (Blackmore et al., 2015)
		# of Calls in Method	(Fursin et al., 2011) (Park et al., 2012) (Namolaru et al., 2010) (Blackmore et al., 2015) (Ashouri et al., 2016)
	Tree-Graph Based Features	CFG Topology	(Pekhimenko and Brown, 2011) (Park et al., 2012) (Kulkarni and Cavazos, 2012) (Namolaru et al., 2010) (Koseki et al., 1997)
		DFG Topology	(Malik, 2010) (Namolaru et al., 2010) (Koseki et al., 1997)
	Dynamic Features	Loop Counts	(Cavazos et al., 2007) (Fursin et al., 2004)
		Hot Code	(Malik, 2010) (Cavazos et al., 2007) (Fursin et al., 2004)
		Cache Hit/Miss Information	(Cavazos et al., 2007) (Ashouri et al., 2016) (Fursin et al., 2004)
		Reactions to Flags	Fursin and Temam (2010)
	Deep Analysis	Abstract Syntax Tree	(Cummins et al., 2017) (Baghdadi et al., 2021)
		Word Embeddings	Peng et al. (2015)

### 2.5 CFG

A control flow graph, *CFG*, is a directed graph with basic blocks as nodes and possible execution orders of those basic blocks as directed edges (Cooper et al., 2012). CFG is like a graphical representation of a program; edges in the graph represent how the execution of the program can potentially proceed, and nodes of the graph represent statements.

#### 2.5.1 Basic block

Each unit in the CFG is called a *basic block*. In a single basic block, a set of operations always execute together (Cooper et al., 2012). Basic blocks have only one entry and one exit point (G. Tea m, 2024).

**FIGURE 4:**
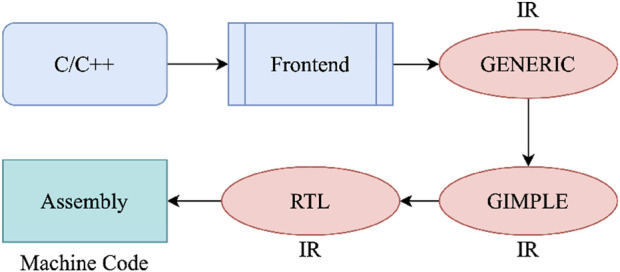
GCC compilation flow: 1) Source Code, 2) Intermediate Representation, and 3) Assembly Code.

CFG of a program can be generated from the compiler’s intermediate representation (IR). GCC pipeline has several IRs ranging from high level (GENERIC, GIMPLE) to low level, close to the assembly (RTL). As seen in Figure 4, when a source code is compiled using GCC, there are several steps before generating the assembly code. In these intermediate steps, GCC generates internal representations, including basic blocks, possible execution patterns, etc., for applying transformations or passes (Gimple, 2024). Therefore, the CFG can be built using RTL and GIMPLE IR of GCC (CFG, 2024).

**FIGURE 5:**
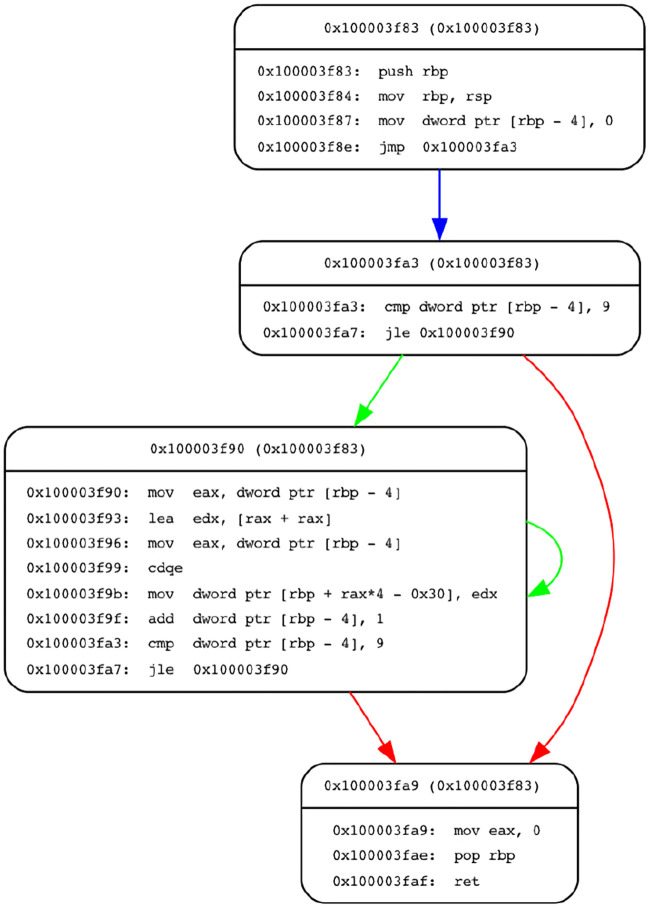
CFG of a simple C program. The CFG is created using ANGR (Wang and Shoshitaishvili, 2017).

An example CFG with its basic blocks and execution order is given as a directed graph shown in Figure 5. In this representation, edges show the execution order, and the edge started from 0x100003f90 block to the 0x100003f90 block shows the for loop.

## 3 Related work

The source code features are directly extracted from the source code itself. One of the earliest studies on source code features was done by Agakov et al. (2006). They tried to automatically focus on the spots likely to improve performance in their work. Even though they used an iterative search algorithm, they created the nearest neighbor (NN) classifier for narrowing the search space. The predictive model then defines a suitable region of interest (ROI) to search. For the NN implementation, they represented the code with 36 different source code features.

In their implementation, the authors used principal component analysis (PCA) to reduce the dimensionality of the dataset. It turns out that most of these raw code features can be used together to create combined features. For example, one can divide the number of load instructions by the number of total instructions to get the memory load ratio (Wang and O’Boyle, 2018). As a result of their analysis, they got a 5D feature vector and used it to train the NN model.

While seeking a solution for the phase ordering problem, Kulkarni and Cavazos worked on extracting per-method-based features in dynamic compilers such as Java (Kulkarni and Cavazos, 2012). They tried to find an order for the optimizations specific to each piece of code.

Namolaru et al. proposed a state-of-the-art model for predicting suitable optimization flags using IR features (Namolaru et al., 2010). In this model, they used GCC’s intermediate representation (G. Team, 2020) to extract static features. They viewed the program as a labeled graph represented by Datalog notation (Namolaru et al., 2010). Furthermore, they proposed to use the relational view of the program by considering several features such as *CFGs*, *Call Graphs*, *Loop Hierarchy*, *DDGs*, etc.

They extracted 56 static features from the relations that comprise the relational representation of the program. Some examples are given as follows:

Milepost uses several features as input to the network and is considered an essential baseline for ML-based approaches in the literature (Blackmore et al., 2017; Malik, 2010; Park et al., 2012; Blackmore et al., 2015).

Koseki et al. proposed using dependence graphs to predict optimal unrolling factors for nested loops (Koseki et al., 1997). They specifically utilized flow dependence, reuse, and output dependencies to predict proper unrolling factors for loops.

Park et al. proposed using CFG for feature extraction in code representation (Park et al., 2012). They used MinIR (Guen et al., 2011) to analyze the intermediate representation (see Section II-E) and extracted a 10-dimensional feature vector for each node in the CFG.

Cavazos et al. used performance counters to extract useful features from programs. Static features are great for finding a correlation between multimedia kernels on embedded processors, but those features performed poorly on more general applications (Cavazos et al., 2007).

Performance counters have been extensively used for performance analysis in explaining program behavior by capturing events. These events can describe several characteristics of the running program, such as cache hits and misses and branch prediction statistics (Cavazos et al., 2007). One of the advantages of performance counters is that they can capture how the target program behaves on specific hardware and avoid the irrelevant information that static code features may bring (Wang and O’Boyle, 2018). Using those counters, authors collected 60 events related to performance, and those events are used as features for an ML model (Cavazos et al., 2007).

Fursin and Temam proposed another dynamic analysis method based on the programs’ reactions to the specific optimization sequences (Fursin and Temam, 2010).

Finding similarities between programs is the challenging part. To find similar programs, they used reactions to some optimization combinations. The response is the performance change when a specific optimization flag is applied to a program (Fursin and Temam, 2010).

Although dynamic analysis can help find handy features, it may be affected by the context switches happening in the system. For instance, a different program running after the population of performance counter registers may alter the measurements and cause using inaccurate values during the dynamic analysis. Therefore, the dynamic analysis may suffer from noise in some environments (Wang and O’Boyle, 2018).

Peng et al. proposed representing programs as vectors for training deep models (Peng et al., 2015). To feed the codes to the deep network, they used language models similar to those used in NLP problems.

Cummins et al. proposed using deep neural network models for feature extraction to find GPU thread coarsening factors (Cummins et al., 2017). Neural networks extract a fixed-size vector that characterizes the entire sequence. This is comparable to the hand-engineered feature extractors used in prior works. Still, it is a learned process that occurs entirely and automatically within the network’s hidden layers.

Similar work is proposed by Zhang et al., where they compare and analyze the similarity between source codes and binary codes for detecting the reused open source codes (Zhang et al., 2021). In their work, they used a famous NLP model, word2vec (Church, 2017), for characterizing the source codes, and using this characterization, they cluster similar applications. They used *clang* compiler and LLVM IR for representing binaries from various architectures in the same format.

Mendis et al. used a data-driven approach to predict the clock cycles a processor takes to execute a block of assembly instructions in a steady state (the throughput). More specifically, they used the assembly code to implement the data-driven technique (Mendis et al., 2019).

Most of the successful efforts in literature use the Milepost project as a baseline and try to outperform it using better features or combining additional information (Blackmore et al., 2017; Malik, 2010; Park et al., 2012; Blackmore et al., 2015).

One of the most successful and interesting efforts among the prior efforts is Park et al.‘s work. As discussed in Section 2, Park et al. proposed using CFG for feature extraction.

They highly depend on hand-crafted feature maps, which require expert input and may change as training data accumulates (Du et al., 2019).

GNN-based solutions also exist in the literature. Allamanis et al. (2018) present a Gated GNN training approach to predict the name of a variable given its usage and to reason about selecting the correct variable. A similar approach is presented by Alon et al. (2018), where authors represent a program using AST, and predict variable names, method names, and data types. Ben-Nun et al. (2018) use the IR of the code and use an RNN architecture along with pre-trained embeddings. They predict performance through compute device mapping, optimal thread coarsening, and algorithm classification. Brauckmann et al. (2020) utilize GNNs for learning predictive compiler tasks for heterogeneous OpenCL mapping. Cummins et al. (2021) propose ProGraML, a language-independent portable program semantic representation, to implement downstream optimization tasks. IR2VEC (VenkataKeerthy et al., 2020) represents programs with distributed embedding based on the IR of the source code. They specifically implement two optimization tasks, namely, heterogeneous device mapping and thread coarsening.

We use GNNs to determine optimal compiler optimization flags through features extracted from the CFG. While prior work has explored the use of GNNs for code optimization, to the best of our knowledge, none of these approaches model basic blocks as node features and control-flow connections as graph edges simultaneously when training a GNN. By explicitly representing both node-level (static) characteristics and edge-level (spatial/structural) relationships, our method captures richer program semantics to guide optimization decisions.

## 4 Our approach

Our main objective is to create a general machine-learning-based automatic code optimization framework that finds suitable optimization flags for a given source code in a reasonable amount of time without executing it. Even though we use the GCC compiler in our implementation, our approach can be applied to any compiler framework with slight modifications. There are several static code analysis methods proposed by lots of researchers in the past (Fursin et al., 2011; Sher et al., 2014; Malik, 2010; Pekhimenko and Brown, 2011; Park et al., 2012; Blackmore et al., 2015; Kulkarni and Cavazos, 2012). These methods relied on extracting hand-crafted features from source code, and those features are fed into machine learning models for learning patterns from source code. Those patterns are then used to find similarities between different applications so that the automatic code optimization framework proposes similar optimization flags to similar programs.

**FIGURE 6:**
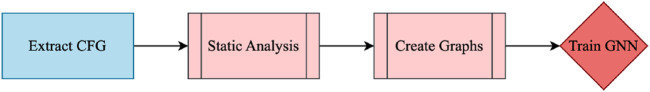
High level view of our approach.

As opposed to the efforts in the literature, we represent source codes using not only the static features but also the programs’ spatial information. After we generate the features, we use neural networks to learn complex patterns of the source codes and propose the best possible optimization flags using the neural network model. Our implementation is composed of four main steps, as shown in Figure 6. Individual steps of the approach can be summarized below:

- Extract the CFG from the intermediate representation.
- Create node features using the static analysis at the basic block level.
- Generate directed graphs using the node features and CFG edges.
- Train a graph neural network for learning patterns from spatial information of the CFG.

We use computationally intensive C benchmark datasets to see the effect of optimization flags. The benchmarks are selected from different domains to understand the capabilities of our model.

One of the most used benchmarks in compiler research is the Polybench benchmark suite due to its clean structure and computationally heavy applications (Pouchet, 2012). The benchmark consists of 30 different applications with different features. In addition to Polybench, we also use cBench (Fursin et al., 2011) as part of our source code pool. Collective Benchmark set, *cBench* was created as an extension to MiBench (Guthaus et al., 2001) and used for training Milepost GCC (Fursin et al., 2011). The dataset has many programs, which all have very long and complex algorithms. Unlike many other works, we used multiple datasets to evaluate our models in different domains.

There are more than 200 optimization flags in GCC. Furthermore, most of them are not binary, requiring arguments. For example, -flto-compression-level flag requires an integer value between 0–19 for defining the compression level of the intermediate language objects (G. Team, 2025). Therefore, training with all the optimization flags would not be feasible. As a result, we select a suitable subset of all the available flags. To do this, we compile our benchmarks with a single optimization flag at a time and record the execution time. We choose the most effective binary flags and use those flags as an initial set. According to the collected results in our benchmark set, we observe that the following flags provide the most significant performance improvements:

-fivopts
-funsafe-math-optimizations
-finline-functions
-fguess-branch-probability
-ftree-loop-optimize
-funroll-all-loops
-ftree-vectorize
-funroll-loops

On average, compiling, running, and analyzing a program in the Polybench set takes roughly 1 min, and for the cBench, that exceeds 5 min (because of the size of applications in the cBench). Even with eight flags, there are $2^{8}=256$ possible binary flag combinations, and generating a dataset for Polybench takes 5 days, and for the cBench, it takes 26 days. Therefore, we work with a good set of optimization flags.

The order of the flags in the optimization set, i.e., phase order, also affects the performance of the optimization as well (Kulkarni and Cavazos, 2012). However, most of the studies in the literature focused on selecting optimization flags and phase ordering problems separately. Therefore, we only focused on selecting optimization flags, and phase order was not considered. Our approach can be extended to include phase ordering as part of the model and optimizations. But this would further complicate the approach, and requires deeper analysis to utilize.

After we choose the flags, we compile and run the programs using the combinations of these optimization flags. We recorded the run time of each possible combination. After creating the dataset, we know which combination takes how long to run.

To observe whether using all the flags gives the best results, we showed all the possible run times using two baselines, namely, the original −O2 and **enhanced** −O2 , meaning that all of the test flags are enabled with the −O2 . We tested our approach against the −O2 optimization level, as it is widely adopted in general-purpose libraries and industrial deployments. We believe that the −O2 provides a fair and representative baseline for most computing environments. The −O3 optimization level is intentionally excluded from our primary comparisons, as it is known to be avoided in certain performance-critical scenarios due to its tendency to increase source code size, which can lead to longer execution times under specific hardware constraints (e.g., CPUs with tiny L1 instruction caches) (Plotnikov et al., 2013; Werner et al., 2020; Georgiou et al., 2018). Consequently, the goal of our proposed method is not to outperform −O3 , but rather to identify more effective optimization sequences on top of −O2 , improving performance without incurring the potential drawbacks associated with enabling all aggressive compiler optimizations.

**FIGURE 7:**
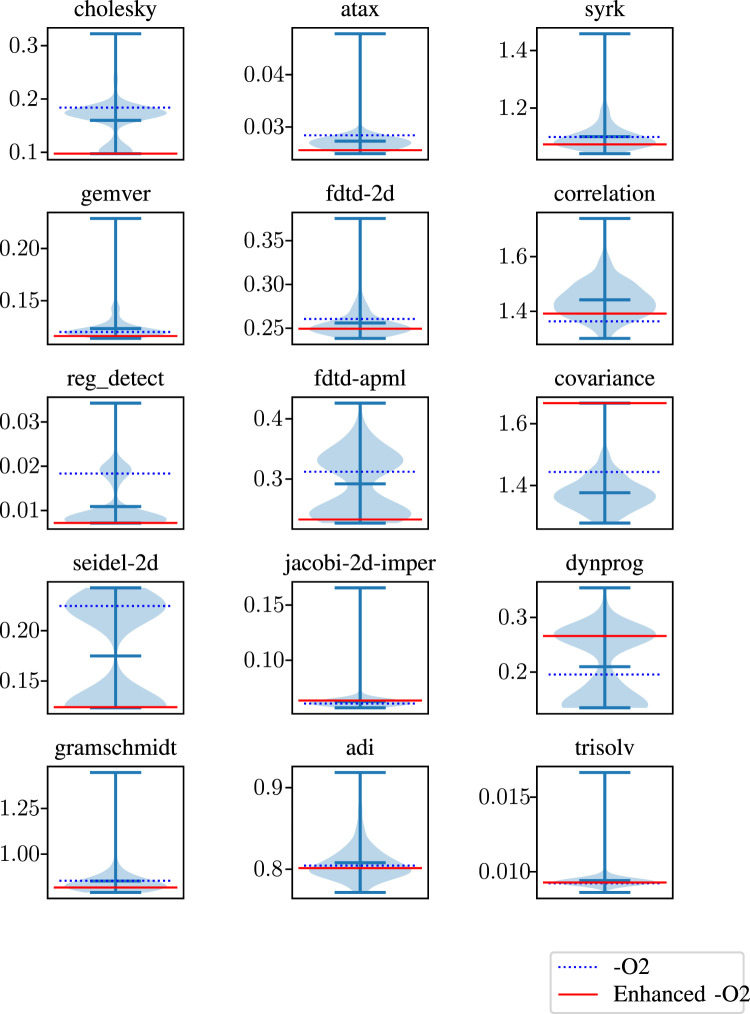
Run times of our optimizations with −O2 and enhanced −O2 baseline for some of the programs in the Polybench benchmark suite.

**FIGURE 8:**
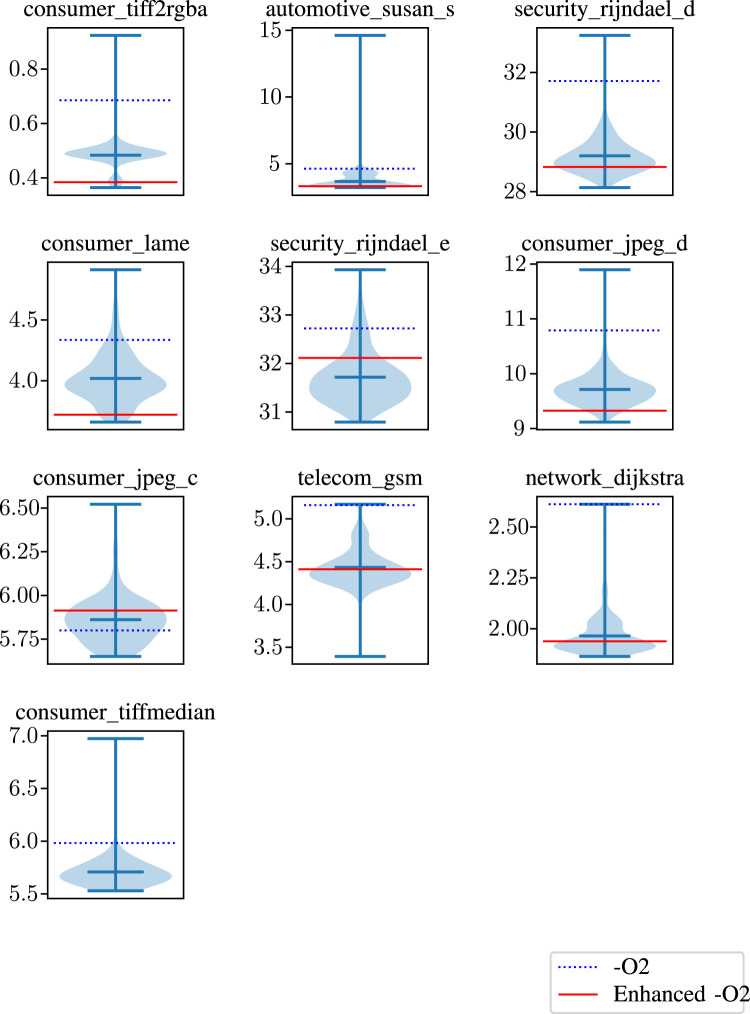
Run times of our optimizations with −O2 and enhanced −O2 baseline for some of the programs in the cBench benchmark suite.

We used violin graphs to visualize all run times and baselines in a single chart. These graphs show the maximum, minimum, and mean run times using three horizontal lines. Also, the shaded area behind these lines offers the distribution of the run times generated by all 256 combinations. Finally, we added two baselines for observing the effect of using no additional flags and all eight flags. The violin graphs in Figures 7, 8 visualize how combinations of our binary flags change at run time. As can be seen from these figures, using all flags does not always yield the best possible result. As discussed in previous sections, some flags affect one another, resulting in performance degradation, such as the *covariance* benchmark in Figure 7. Therefore, our model tries to find the best possible combination instead of using all flags.

Our approach analyzes both source code features and CFG for spatial information. As mentioned before, to extract data from CFG, we need to examine the IR or assembly code directly. Analyzing IR requires some external frameworks with limited capabilities, such as MinIR (Guen et al., 2011). To integrate into different compilation pipelines, we did not modify GCC by writing compiler passes. Instead, we analyze the assembly code by directly working on binary. Therefore, one of the essential features of our framework is that it does not require modifications in the existing compiler or installing third-party analyzer tools.

For analyzing the assembly output of the GCC compiler, we used the ANGR library (Wang and Shoshitaishvili, 2017). ANGR is an open-source binary analysis library created for Python, which allows us to perform static analysis on binary outputs. We use the following static features to extract application characteristics.

**FIGURE 9:**
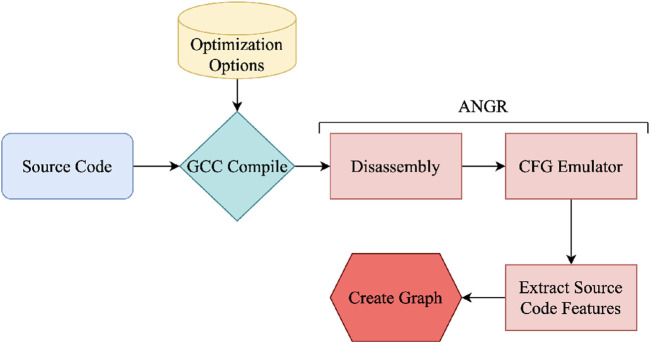
Our source code analysis framework to create the graph to be used in our GNN model.

Our source code analysis framework is given in Figure 9. First, we compile the source code using a GCC compiler with the desired optimization options. Then, the binary executable file is fed into our framework. We first use the disassembly function of ANGR to get the assembly instructions of the executable file. Then, we extract the CFG of the source code using the CFGEmulated function, which gives the basic block information (such as addresses, instructions inside these basic blocks, and edges between them) as shown in Figure 5.

After we get the CFG information, we extract the features from the basic blocks. Since we have the instructions inside the basic blocks, we generate node features using the static source code analysis. The node features help the GNN to learn the spatial information (the edges between nodes) and the structure of the node itself. The node features that we used are as follows:

- Number of arithmetic operations: This feature is the number of total arithmetic operations inside the basic block.
- Number of floating point arithmetic operations: Total number of floating point operations. We added this separately since floating point operations consume more time than the other arithmetic operations.
- Number of memory operations: Operations that require memory access like MOV, POP, PUSH, etc.
- Number of floating point memory operations: Floating point memory operations such as MOVSS, etc.
- Number of compare operations: Number of comparison operations that may lead to jumps and branches.
- Number of jump operations: Number of total jump operations that may lead to branches.
- Number of function operations: Total number of function calls inside the basic block.
- Number of return operations: Indicator of whether the basic block returns anything.
- Number of register accesses: Number of total register accesses like RAX, RES, R11, etc.
- Number of total instructions: Number of all instructions inside the basic block.
- Number of predecessors of the current basic block: This parameter shows the number of branches before that basic block. We use this information as an indicator of loops.
- Number of successors of the current basic block: This parameter shows the number of branches after that basic block if the basic block is a branching one. We use this information as an indicator of loops.
- Loop depth: How many loops cover the current basic block?

Using basic blocks as nodes, basic block features as node features, and connections between those nodes as edges, we create a directed graph for each output that we generate using the combinations of our binary flags.

We have 128× [ NumberofApplications ) $\left(2^{7}=128\right)$ labeled graphs for a single optimization flag. Also, to prevent mislabeling due to slight differences in run time, we check whether the added flag improves the performance more than 10% of the initial run time. This way, we prevented the noises generated by the machine’s state at run time.

After we label the graphs, we normalize the features of the nodes and create a data loader for the training. However, since some of the flags have unbalanced labels, before training, we balance the labels 50% - 50% by oversampling. To increase the number of graphs in the small set, we randomly choose more samples until the two classes have equal samples.

## 5 Graph neural network (gnn) model

GNN architecture consists of graph convolution layers, linear layers, and non-linear activation functions. We tested different network configurations with various parameters to measure the accuracy and the loss. The parameters that we tuned in the GNN model are as follows:

**Number of Layers:** A total number of graph convolution layers in the network. As the complexity of the task increases, the number of layers can also be increased. However, using too many layers may lead to loss of information, known as *vanishing gradient problem* as well (Hochreiter, 1998). Therefore, this parameter should be tuned carefully according to its complexity. A *layer* contains a graph convolution module, a linear module, and a non-linear activation function.

**FIGURE 10:**
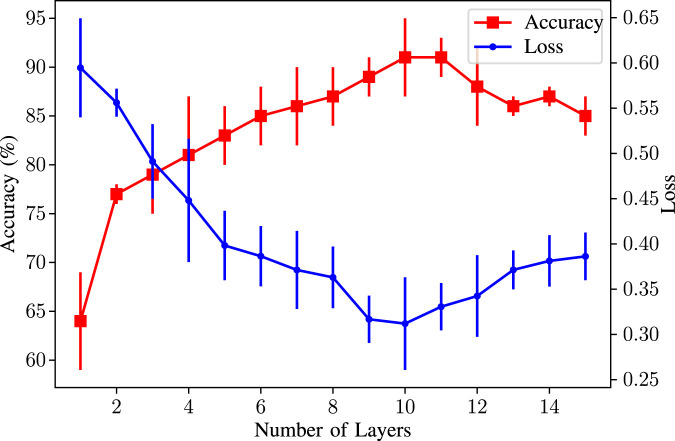
Accuracy/Loss vs. number of layers used in the GNN model.

To find the optimum number of layers, we tried different layers from 1 to 15. While changing the number of layers, we kept all other parameters fixed so that we could observe the change only caused by the number of layers. Based on our experiments, 10 is the optimum number of layers. The changes in the average accuracy and the average loss with error bars indicating the different values in the different cross-validation sets are given in Figure 10. As can be seen, 10 is our dataset’s optimum number of layers. Although more layers mean more features to learn and the node feature information can be propagated to other nodes, practical observations show that using more layers may drop the performance (Zhou et al., 2021). This is caused by *over smoothing* of the node features in deep networks (Hochreiter, 1998).

**FIGURE 11:**
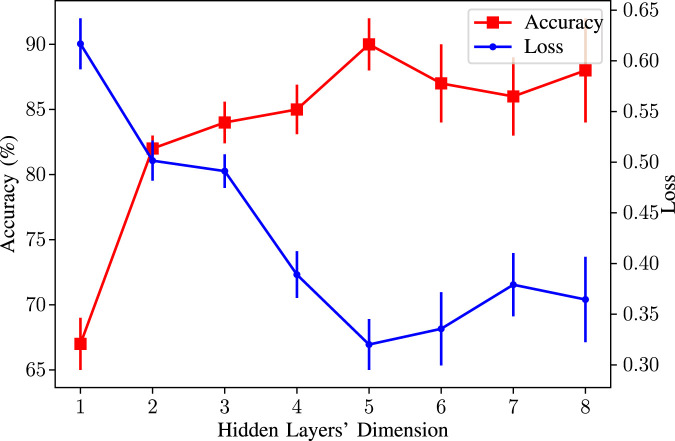
Accuracy/Loss vs. hidden layers’ dimension used in our GNN model.

**Hidden Layers’ Dimension:** The dimension of the output of the hidden units. For this parameter, we tried different values from 1 to 8. Our experiments show that the optimum dimension for our dataset is 5. We keep all other parameters fixed and change only the dimension of the hidden units. The changes in average accuracy and the loss given in Figure 11 indicate that the optimum dimension for our dataset is 5.

**Neighbor Aggregation Type:** Aggregation type of the neighboring nodes. This parameter defines how to combine information on neighboring node features. We use three different aggregation types: The maximum of features of the neighboring nodes *(max)*, the mean of the features of the neighboring nodes *(mean)* or the sum of the features of the neighboring nodes *(sum)*.

**Graph Pooling Type:** Aggregation type of the information between nodes (Wang and Ji, 2020). It is used for converting the graph to a 1D vector at the end. Since pooling is used to obtain graph representations from node representations, this parameter defines how the network should combine all the node information (Wang and Ji, 2020). We tried three possible pooling types for the graph pooling operation to find the best pooling type.

**FIGURE 12:**
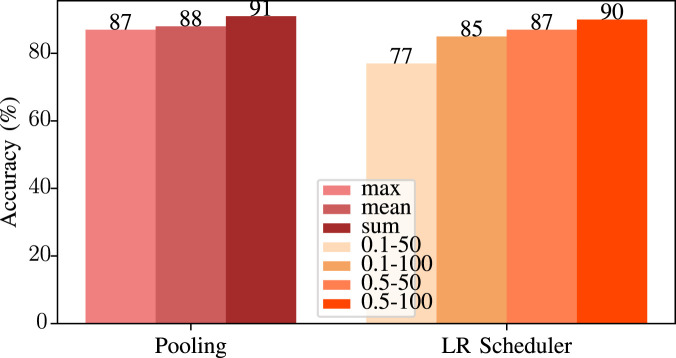
Accuracy vs. pooling types and LR scheduler parameters used in our GNN model.

Results for all pooling types are given in Figure 12. As can be seen, we are using *sum* as graph pooling is the optimum choice.

In networks where the number of neighbor nodes varies greatly, using sum as neighbor aggregation can lead to problems such as nodes with high degrees may have much larger aggregation than nodes with low degrees (Wang and Ji, 2020). In our case, since the number of neighbors is close in all nodes in the graphs that we generated from the source codes, instead of averaging them or taking the maximum of the node features, using *sum* as pooling type resulted in the best accuracy. This is expected since the program spends most of its time on the basic blocks with the heaviest load. This follows from the fact that our features grow when the block is heavier with a higher number of nodes, instructions, etc. Therefore, as we sum all the information from the convolution operation, the aggregated data shows the computationally heavy parts of the source code, which allows the network to focus on the most time-consuming aspects.

**LR Scheduler:** Learning rate (LR) scheduler is used for adjusting the learning rate during the training. To see the effects of the LR scheduler, we tried two different Gamma parameters with two different step sizes. In the best case, the LR scheduler step size is set to 100, and the gamma is set to 0.5. Since we are reducing LR 10 times, using 0.1 would result in a tiny LR, nearly stopping the learning process. As an initial learning rate, we choose $10^{-4}$ since this value resulted in the best results in our fine-tuning runs.

While using a higher learning rate achieves faster training, keeping it too large may result in divergence. In such a scenario, the network may miss what is essential. On the other hand, using a minimal LR results in convergence but leads to very long training times. Therefore, the LR scheduler creates an adaptive learning rate. LR starts high and after some time, it becomes smaller. *Step size* specifies the number of times the entire dataset should pass through the network (number of epochs) to decrease the learning rate. For example, step size 50 means in every 50 generations, LR will be reduced.

**LR Scheduler - *Gamma*:** This parameter specifies the ratio of the decrease in the LR. For instance, gamma 0.1 and step size 50 means LR will be reduced to one-tenth in every 50 epochs. If Gamma is chosen too large, the learning rate may be reduced too quickly, leading to very slow convergence, while using it very small may not decrease the learning rate effectively, so the network may not learn the small details.

All possible four pairs are given in Figure 12. In this graph, values in the y-axis are the gamma and step size pairs, where the first value is the gamma and the second value is the step size for the LR scheduler.

**Weight Initialization Method:** The weight initialization is crucial for the network’s convergence (completing the learning phase). If the weights are correctly initialized, the learning process will be much shorter and easier (Kumar, 2017). Therefore, we tested our network with and without the Xavier weight initialization method. Results showed that Xavier weight initialization increased the accuracy from 82% to 91% .

Properly initialized weights increase the performance of the network dramatically (Kumar, 2017).

This is because Xavier prevents weights from exploding or vanishing by keeping the variance the same across every layer in the network (Kunin, 2019).

For instance, although the difference between 1.1 and 0.9 is just 0.2, in a network with 50 layers, when the weights are multiplied repeatedly, $1.1^{50}=117930$ while $0.9^{50}=0.00515$ . The difference shows the importance of weight initialization, which we tried avoiding using Xavier (Kunin, 2019).

**Loss Function:** We trained our model for 500 epochs and recorded the accuracy and the loss values. As a loss function, we used *Cross Entropy Loss* since we have a binary classification task. Cross entropy measures the difference between the probability distribution of a machine learning classification model and the predicted distribution as given in Equation 1, where p is the predicted probability, y is the ground truth, and l is the loss (Maheskar, 2019).

$$l=-\left(y\times log\left(p\right)+\left(1-y\right)\times log\left(1-p\right)\right)$$ (1)

To fine-tune the model, we split our dataset into the training, validation, and test sets. We used the 5-fold cross-validation technique for fine-tuning and created validation sets of 1,000 graphs to calculate the accuracy and loss during the fine-tuning.

**Network Optimizer:** We used *Adaptive Moment Estimation (Adam)* as an optimizer in our network. Adam optimizer is used in many ML applications using cross-entropy loss, and it is proven to be a successful algorithm for convergence where cross-entropy loss is used (Bock and Weiß, 2019).

## 6 Evaluation

We evaluate our scheme by measuring the compiled application’s run time and comparing it against two different baselines. The first is −O2 , and the second is *enhanced* −O2 . We turn all the tested flags on in the enhanced −O2 case. Therefore, we use both extremes, where we have all flags enabled and all flags disabled on top of −O2 .

### 6.1 Setup

We have used two benchmark datasets, Polybench and cBench. Polybench is a collection of benchmarks containing different numerical computations. These computations are extracted from domains like linear algebra, image processing, statistics, physics simulation, etc. (Abella-González et al., 2021). The cBench dataset contains real-world applications with high computational requirements. It contains six categories (Guthaus et al., 2001), namely, Automotive and Industrial, Network, Security, Consumer Devices, Office Automation, and Telecommunications.

The compilation, source code analysis, and graph generation operations are done using a MacBook Pro (macOS Big Sur v11.0.0.1) with a 3.1 GHz Dual-Core Intel Core i7 CPU and 16 GB of memory. GNN training operations are done on a workstation with an Intel Core i9 CPU, 128 GB of memory, and NVIDIA RTX 3090 GPU. For compilation, we used gcc v11.2.0 (G. Team, 2022). Our Python scripts are written using Python 3.9. For binary analysis we used angr v9.1.109 (Wang and Shoshitaishvili, 2017). For GNN training operations, we have used torch v1.9.1 and dgl v0.6.1 (Wang et al., 2019) libraries.

### 6.2 Accuracy results

#### 6.2.1 Effectiveness of GNN model

We compared three different classifier models with our method to see how using a GNN model affects performance. We tested a 1-nearest neighbor classifier, similar to the one used in Milepost, and also trained a Deep Neural Network using our training dataset. To fit a 1-nearest neighbor classifier, we flattened all of our node features and summed them as if we had a single feature vector for each program. For instance, instead of extracting the number of arithmetic operations for each basic block, we now have a total number of operations in the whole program as a feature. Also, to observe the effect of spatial features, we trained a deep neural network model that also has ten layers. We again flattened the graphs and created a single feature vector for each program. Our results show that using a deep neural network provides an 81% accuracy. The results show that the GNN model outperformed both nearest neighbor and neural network models with the help of the spatial information that we extracted.

#### 6.2.2 Results for single-flag experiments

We trained our network with a single flag for these experiments. Labels used in this experiment are as follows:

• 0: The flag did not reduce the run time of the graph.
• 1: The flag reduced the run time of the graph.

**FIGURE 13:**
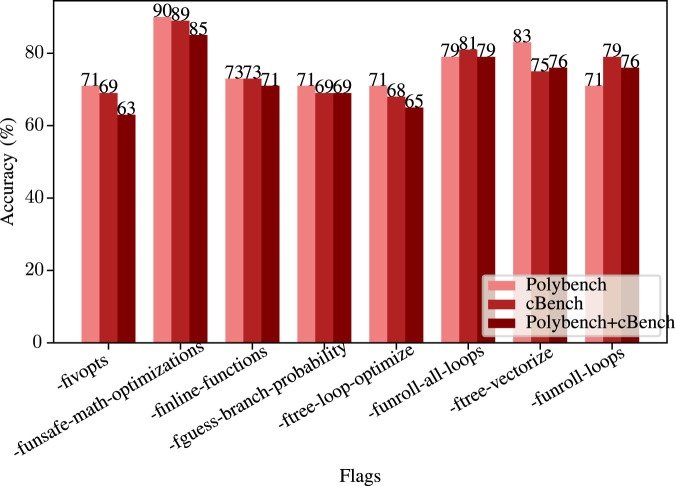
Single flag accuracy when trained with different benchmark sets.

First, we trained our network using the Polybench dataset, and tested using individual flags also in this dataset. Then, we did the same for the cBench dataset. So, the training sets and test sets are from the same benchmark suite. The accuracy results for these tests are given in Figure 13.

When we compare the accuracy results of the Polybench set, we observe that the best performance is achieved by the -funsafe-math-optimizations flag. The Polybench set consists of applications in domains like linear algebra and statistics. Therefore, the result shows that our model learned the patterns of math applications from the Polybench dataset, resulting in the best performance for -funsafe-math-optimizations. The second best flag, -ftree-vectorize, also supports our discussion. Moreover, since the Polybench set consists of matrix multiplication algorithms, we can say that our model learned when to unroll loops since we have 79% accuracy for the -funroll-all-loops as well.

The cBench results show that the best performance is achieved again with the -funsafe-math-optimizations. Since both benchmark sets target CPUs, they contain computationally intensive algorithms. Therefore, our model mostly focused on those math applications, which is why it performs best with -funsafe-math-optimizations. However, for cBench set, -funroll-all-loops performs better than -ftree-vectorize. This shows that applications with data-intensive operations like media applications in cBench helped our model learn patterns for -funroll-all-loops flag.

When we train our model using both benchmark sets combined in a single pool, we observe a 4% accuracy drop on average for all the flags, as seen in the last bars of Figure 13. This is also expected because the network now tries to learn from small and large programs. Also, training in cross-domains is a challenging task as well (Wang et al., 2021). To strictly prevent data leakage, we employed a Leave-One-Out validation strategy, ensuring that the model never encountered the specific program being tested during the training phase. The slight reduction in accuracy on the combined Polybench and cBench dataset is attributable to the distinct domain gap between the two suites. Polybench consists of small mathematical kernels (approx. 100 basic blocks), whereas cBench contains complex system applications (often 1,000+ basic blocks). Despite this increased complexity, our model demonstrated strong generalization capabilities in the cross-domain experiments; specifically, the model trained on cBench successfully optimized the unseen Polybench suite, achieving an average speed-up of 8.44% over −O2 . We believe this confirms the model’s ability to learn and apply optimization patterns across differing domains.

Results also show that our network performed best on the Polybench set. This is relatively expected because Polybench includes simpler programs when compared to cBench. For instance, while a typical Polybench program contains around 100 nodes (basic blocks), a typical cBench program has more than 1,000 nodes (even sometimes 6,000 nodes). Therefore, some of the cBench programs are complex, so it is hard to predict from thousands of nodes, which results in a nearly 2% decrease in accuracy.

However, for some flags, the results are better for cBench. The reason for this behavior may be related to the domain of the datasets. While the Polybench contains mainly math applications (2D matrix multiplications, image processing tasks, etc.), the cBench contains sorting, zipping, and encryption algorithms. Therefore, while -ftree-vectorize, the flag that enables auto-vectorization (vectorizes the convolution loops for faster execution) (G. Team, 2011), performed significantly better on Polybench, -funroll-all-loops, a flag that speeds up the run time by unrolling all loops (G. Team, 2025), performed better on the cBench.

**FIGURE 14:**
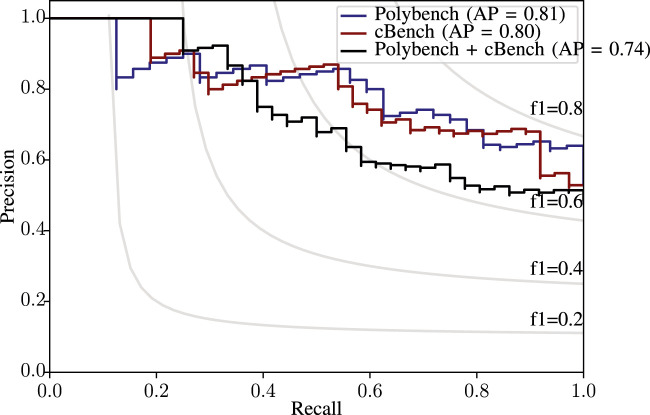
Precision-recall curves for single flag tests.

Our models’ average precision-recall (PR) curves are given in Figure 14. The PR curves show that for Polybench and cBench models, the average precision value is very close, and we obtain an F1 score approximately equal to 0.8 for both sets. The slight difference between the performance of Polybench and cBench is also observed here. Figure 14 shows the PR curve for both models. The 6% drop in average precision shows that the models performed relatively poorer than the single dataset models.

#### 6.2.3 Results for two-flag experiments

To perform a deeper analysis, we evaluate the model’s inference performance with more than one flag. For this test, we use two flags in the model, and we try to find the best choice among four possible scenarios:

• 0: Using no flags results in the best run time.
• 1: Using only the first flag results in the best run time.
• 2: Using only the second flag results in the best run time.
• 3: Using both flags results in the best run time.

**FIGURE 15:**
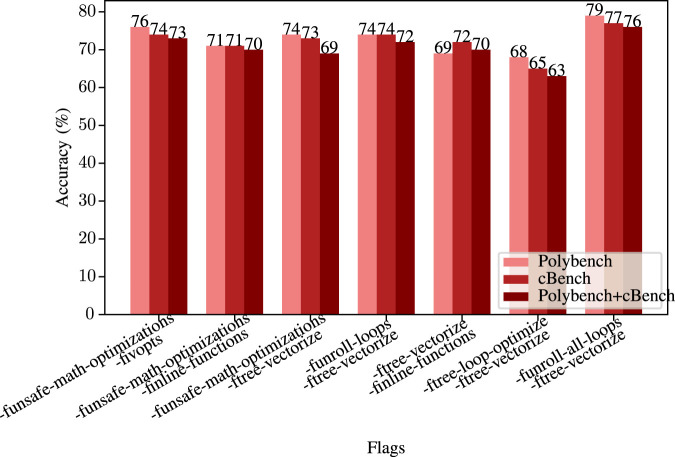
Two flag accuracy comparison with Polybench, cBench, and both benchmarks.

We labeled our graphs according to these choices with four labels. There are 28 possible two-flag combinations for eight flags, and we chose only 7 of them at the beginning for a proof of concept. After labeling, we train the model for Polybench, cBench, and both, as we did in the single flag tests. The accuracy results on test sets are given in Figure 15.

Instead of deciding whether a particular flag will reduce the run time, we are now deciding if we should use the first, second, or both. Therefore, the model’s primary purpose here is to find the best possible solution instead of just reducing the run time.

According to the accuracy results for two flags on the Polybench set, we achieve a 73% accuracy on average. Although this is not as successful as the single flag test, it is acceptable since the model has four labels.

The flag correlation between two flag results’ accuracy and single flag results has an interesting pattern. More specifically, as can be seen in Figure 15, while two flag accuracy for -ftree-loop-optimize and -ftree-vectorize is around 65% , the single flag accuracy for -ftree-loop-optimize is around 68% , and -ftree-vectorize is around 75% . The two-flag accuracy is close to the minimum single-flag accuracies among the pairs. We observe similar patterns for all the pairs.

### 6.3 Performance results

We conducted tests using our benchmark sets to see how our model performs in real use cases. To measure the performance of our methodology, we first exclude one program from the dataset, then we train the model using the rest of the programs in the dataset. The last step is to measure the performance of the excluded program.

**FIGURE 16:**
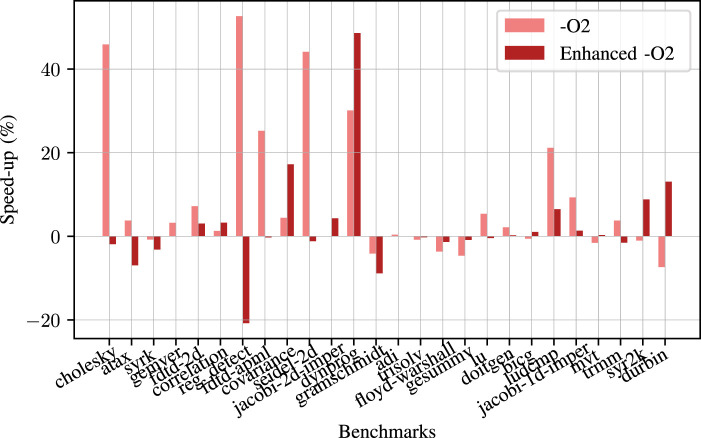
Speed-up for each benchmark in Polybench benchmark suite compared to −O2 and enhanced −O2 baselines.

Figure 16 shows the speed-up percentages for each benchmark in the Polybench dataset. Our approach results in faster run times for 15 out of 26 programs when compared with −O2 . The average speed-up compared to −O2 for the Polybench dataset is **9.06%** with a maximum of **52%**. In the worst case, we generate a 7% slow down compared to the −O2 run times.

When we compare our results with the enhanced −O2 where all the tested flags are enabled, it turns out that we get faster run time in 10 out of 26 programs. Our model’s average speed-up for the Polybench dataset is **2.33%** with a maximum of **48.6%**. In the worst case, we generate a 20% slowdown compared to the case where all the tested flags are enabled. We observe the worst results for *reg_detect* benchmark because it has several loops with undefined loop counts. Furthermore, since the loop characteristics highly depend on the input data, the model could not predict the best flags for this benchmark.

For the 58% of the benchmarks in Polybench, our predictions are faster than −O2 with an average **16.93%** speed-up. Moreover, our predictions are faster than enhanced −O2 for 39% of all the benchmarks with an average of **10.1%**.

**FIGURE 17:**
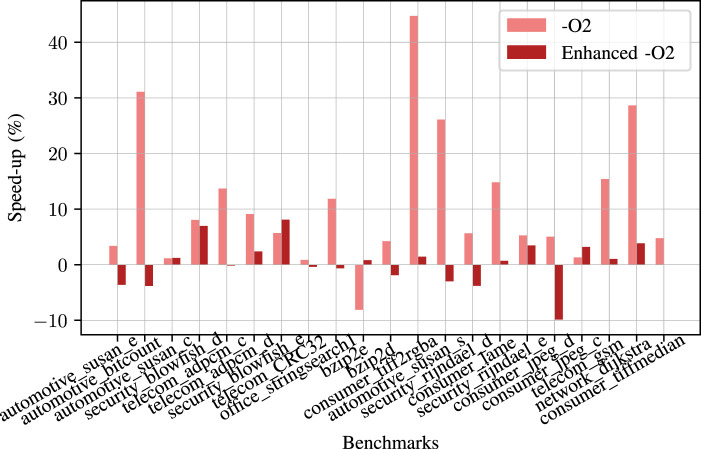
Speed-up for each benchmark in cBench benchmark suite compared to −O2 and enhanced −O2 baselines.

The speed-up results for the cBench set are given in Figure 17. In this benchmark set, we get better run time results than −O2 for 19 out of 21 programs. The average speed-up for the cBench dataset is **11.07%** with a maximum of **44%**. In the worst case, our approach results in an 8% slow down compared to the −O2 run time. We observe this result for the *consumer_jpeg_d* benchmark since this benchmark has several loops in which their loop counts highly depend on the input data, as we observed in *reg_detect* in our Polybench set.

When we compare our results with the enhanced −O2 , we get better run times for 9 of 21 benchmarks in the cBench dataset. The average speed-up that we get is **0.28%** with a maximum of **8.01%**, and in the worst case, our approach results with a **9.87%** slow down compared to the all tested flags enabled case.

For 90% of the benchmarks in cBench, our results perform faster than −O2 with an average speed-up of **12.21%**. Moreover, our predictions are faster than the enhanced −O2 for 43% of all the benchmarks with an average speed-up of **3.1%**.

The single domain results show that, on average, our model generates better run times when compared to −O2 and enhanced −O2 . In the cases where our approach generated faster run time than the baselines, our average speed-up is better for the Polybench set. This is expected since benchmarks in Polybench are simpler programs than the cBench. Although our results generated faster results for more programs in cBench, the average speed-up is higher in the Polybench suite.

Polybench mainly includes applications from domains such as linear algebra and statistics, where mathematical operations dominate. In contrast, cBench consists of real-world, computationally intensive applications from diverse domains. We also evaluated our approach in a mixed-domain setting, training on one benchmark and testing on the other, where accuracy decreased by only 4%. This may be viewed as a limitation of the approach; however, the observed drop is acceptable given the substantial differences between the domains. These results suggest that the approach can generalize to other domains, as key program characteristics are captured by the selected benchmarks. Performance could be further improved by training on a broader and more diverse set of applications, or by tailoring training to a specific target domain when specialization is required.

In future work, to improve the accuracy and performance of the results, we plan to use *transfer learning*. When the training data is expensive or hard to collect, a model trained with easily collected data can be transferred to another domain by introducing less with the new data (Weiss et al., 2016). Therefore, we can prepare a base model using standard benchmarks like cBench and Polybench, train the base model using fewer data from specific domains, and create a particular model for different domains.

## 7 Conclusion

There are many efforts focused on finding an optimization sequence automatically by analyzing source code. In this work, we implement a combined approach that utilizes static, spatial, and deep analysis methods. To extract all of the information from the source code, we analyzed the source code at the basic block level. We extracted static code features from basic blocks and created a feature vector for each basic block in the source code using the CFG, which contains essential spatial features like loops, jumps, calls, etc. We created a GNN model and trained the model using the graphs we created. Unlike the SVM kernels, GNN learned much more complex patterns from the graphs thanks to its layered structure. As a result, we implement a GNN model that predicts an optimization sequence for a program without running it. Our results show that the GNN model is not only capable of proposing suitable optimization flags in a single domain, but it is also capable of providing suitable optimization flags on different domains as well. Therefore, we can conclude that static and spatial features successfully represent code, and GNN models can learn complex patterns from source code.

To improve the cross-domain results, we plan to use *transfer learning*. When the training data is expensive or hard to collect, a model trained with easily collected data can be transferred to another domain by introducing less with the new data (Weiss et al., 2016). Therefore, we can prepare a base model using standard benchmarks like cBench and Polybench, train the base model using fewer data from specific domains, and create a particular model for different domains.

Our approach currently works with eight flags, which is orthogonal to the approach itself. Even though this set of flags provides a considerable coverage, this set can be scaled for different application domains that can potentially utilize these flags. Another limitation in our work is about the phase order, which can potentially affect the performance. Our approach can be extended to include phase ordering as part of the model and optimizations.

## Data Availability

The original contributions presented in the study are included in the article/supplementary material, further inquiries can be directed to the corresponding author.

## References

[B1] Abella-González M. Á. Carollo-Fernández P. Pouchet L.-N. Rastello F. Rodríguez G. (2021). “Polybench/python: benchmarking python environments with polyhedral optimizations,” in Proceedings of the 30th ACM SIGPLAN international conference on compiler construction, 59–70.

[B2] Agakov F. Bonilla E. Cavazos J. Franke B. Fursin G. O’Boyle M. F. (2006). “Using machine learning to focus iterative optimization,” in International Symposium on Code Generation and Optimization (CGO’06), New York, NY, USA, 26-29 March 2006 (IEEE), 11.

[B3] Allamanis M. Brockschmidt M. Khademi M. (2018). Learning to represent programs with graphs. Available online at: https://arxiv.org/abs/1711.00740.

[B4] Alon U. Zilberstein M. Levy O. Yahav E. (2018). A general path-based representation for predicting program properties. SIGPLAN Not. 53 (4), 404–419. 10.1145/3296979.3192412

[B5] Ashouri A. H. Mariani G. Palermo G. Park E. Cavazos J. Silvano C. (2016). Cobayn: compiler autotuning framework using bayesian networks. ACM Trans. Archit. Code Optim. (TACO) 13 (2), 1–25. 10.1145/2928270

[B6] Baghdadi R. Merouani M. Leghettas M.-H. Abdous K. Arbaoui T. Benatchba K. (2021). A deep learning based cost model for automatic code optimization. Proc. Mach. Learn. Syst. 3, 181–193.

[B7] Ben-Nun T. Jakobovits A. S. Hoefler T. (2018). “Neural code comprehension: a learnable representation of code semantics,” in Proceedings of the 32nd International Conference on Neural Information Processing Systems (NIPS'18), Red Hook, NY: Curran Associates Inc., 3589–3601.

[B8] Blackmore C. Ray O. Eder K. (2015). A logic programming approach to predict effective compiler settings for embedded software. Theory Pract. Log. Program. 15 (4-5), 481–494. 10.1017/s1471068415000174

[B9] Blackmore C. Ray O. Eder K. (2017). Automatically tuning the gcc compiler to optimize the performance of applications running on embedded systems. arXiv preprint arXiv:1703.08228.

[B10] Bock S. Weiß M. (2019). “A proof of local convergence for the adam optimizer,” in 2019 International Joint Conference on Neural Networks (IJCNN), Budapest, Hungary, 14-19 July 2019 (IEEE), 1–8.

[B11] Branco D. Henriques P. R. (2015). “Impact of gcc optimization levels in energy consumption during c/c++ program execution,” in 2015 IEEE 13th International Scientific Conference on Informatics., Poprad, Slovakia, 18-20 November 2015 (IEEE), 52–56.

[B12] Brauckmann A. Goens A. Ertel S. Castrillon J. (2020). “Compiler-based graph representations for deep learning models of code,” in Proceedings of the 29th international conference on compiler construction, ser. CC 2020 (New York, NY, USA: Association for Computing Machinery), 201–211. 10.1145/3377555.3377894

[B13] Cavazos J. Fursin G. Agakov F. Bonilla E. O’Boyle M. F. Temam O. (2007). “Rapidly selecting good compiler optimizations using performance counters,” in International Symposium on Code Generation and Optimization (CGO’07), San Jose, CA, USA, 11-14 March 2007 (IEEE), 185–197.

[B14] CFG (2024). Control flow graph (cfg). Available online at: https://gcc.gnu.org/onlinedocs/gccint/Control-Flow.html (Accessed March 4, 2026).

[B15] Church K. W. (2017). Word2vec. Nat. Lang. Eng. 23 (1), 155–162. 10.1017/s1351324916000334

[B16] Cooper K. D. Torczon L. (2012). “Chapter 5 - intermediate representations,” in Engineering a compiler. Editors Cooper K. D. Torczon L. second edition (Boston: Morgan Kaufmann), 221–268. Available online at: https://www.sciencedirect.com/science/article/pii/B9780120884780000050 (Accessed March 4, 2026).

[B17] Cummins C. Petoumenos P. Wang Z. Leather H. (2017). “End-to-end deep learning of optimization heuristics,” in 2017 26th International Conference on Parallel Architectures and Compilation Techniques (PACT), Portland, OR, USA, 09-13 September 2017 (IEEE), 219–232.

[B18] Cummins C. Fisches Z. V. Ben-Nun T. Hoefler T. O’Boyle M. F. P. Leather H. (2021). “ProGraML: a graph-based program representation for data flow analysis and compiler optimizations,” in Proceedings of the 38th International Conference on Machine Learning, Proceedings of Machine Learning Research. Editors Meila M. Zhang T. (PMLR), 139, 2244–2253. Available online at: https://proceedings.mlr.press/v139/cummins21a.html (Accessed March 4, 2026).

[B19] Du S. S. Hou K. Salakhutdinov R. R. Poczos B. Wang R. Xu K. (2019). Graph neural tangent kernel: fusing graph neural networks with graph kernels. Adv. Neural Information Processing Systems 32.

[B20] Fursin G. Temam O. (2010). Collective optimization: a practical collaborative approach. ACM Trans. Archit. Code Optim. (TACO) 7 (4), 1–29. 10.1145/1880043.1880047

[B21] Fursin G. O’Boyle M. F. Temam O. Watts G. (2004). A fast and accurate method for determining a lower bound on execution time. Concurrency Comput. Pract. Exp. 16 (2-3), 271–292. 10.1002/cpe.774

[B22] Fursin G. Kashnikov Y. Memon A. W. Chamski Z. Temam O. Namolaru M. (2011). Milepost gcc: machine learning enabled self-tuning compiler. Int. Journal Parallel Programming 39 (3), 296–327. 10.1007/s10766-010-0161-2

[B23] G. Team (2024). Basic blocks. Available online at: https://gcc.gnu.org/onlinedocs/gccint/Basic-Blocks.html (Accessed March 4, 2026).

[B24] G. Team (2011). Auto-vectorization in gcc. Available online at: https://gcc.gnu.org/projects/tree-ssa/vectorization.html (Accessed March 4, 2026).

[B25] G. Team (2020). Rtl - intermediate representation called register transfer language. Available online at: https://gcc.gnu.org/onlinedocs/gcc-3.4.6/gccint/RTL.html (Accessed March 4, 2026).

[B26] G. Team (2022). Gcc 11 release series. Available online at: https://gcc.gnu.org/gcc-11/changes.html (Accessed March 4, 2026).

[B27] G. Team (2025). Options that control optimization. Available online at: https://gcc.gnu.org/onlinedocs/gcc-4.5.2/gcc/Optimize-Options.html (Accessed March 4, 2026).

[B28] Georgiou K. Blackmore C. Xavier-de Souza S. Eder K. (2018). “Less is more: exploiting the standard compiler optimization levels for better performance and energy consumption,” in Proceedings of the 21st international workshop on software and compilers for embedded systems, 35–42.

[B29] Gimple (2024). Gimple. Available online at: https://gcc.gnu.org/onlinedocs/gccint/GIMPLE.html (Accessed March 4, 2026).

[B30] Guen J. Guillon C. Rastello F. (2011). “Minir: a minimalistic intermediate representation,” in Workshop on intermediate representations (WIR’11) (Chamonix, France).

[B31] Guthaus M. R. Ringenberg J. S. Ernst D. Austin T. M. Mudge T. Brown R. B. (2001). “Mibench: a free, commercially representative embedded benchmark suite,” in Proceedings of the fourth annual IEEE international workshop on workload characterization. WWC-4 Cat. No. 01EX538, Austin, TX, USA, 02-02 December 2001 (IEEE), 3–14.

[B32] Hochreiter S. (1998). The vanishing gradient problem during learning recurrent neural nets and problem solutions. Int. J. Uncertain. 6 (02), 107–116. 10.1142/s0218488598000094

[B33] Koseki A. Komastu H. Fukazawa Y. (1997). “A method for estimating optimal unrolling times for nested loops,” in Proceedings of the 1997 International Symposium on Parallel Architectures, Algorithms and Networks I-SPAN’97, Taipei, Taiwan, 20-20 December 1997 (IEEE), 376–382.

[B34] Kulkarni S. Cavazos J. (2012). “Mitigating the compiler optimization phase-ordering problem using machine learning,” in Proceedings of the ACM international conference on object oriented programming systems languages and applications, 147–162.

[B35] Kumar S. K. (2017). On weight initialization in deep neural networks. arXiv Preprint arXiv:1704.08863.

[B36] Kunin K. (2019). Initializing neural networks. Available online at: https://www.statistics.com/glossary/loss-function/(Accessed August 12, 2022).

[B37] LLVM (2024). Llvm’s analysis and transform passes. Available online at: https://llvm.org/docs/Passes.html (Accessed March 4, 2026).

[B38] Maheskar S. (2019). What is cross entropy loss? Available online at: https://wandb.ai/sauravmaheshkar/cross-entropy/reports/(Accessed August 12, 2022).

[B39] Malik A. M. (2010). “Spatial based feature generation for machine learning based optimization compilation,” in 2010 Ninth International Conference on Machine Learning and Applications, Washington, DC, USA, 12-14 December 2010 (IEEE), 925–930.

[B40] Mendis C. Renda A. Amarasinghe S. Carbin M. (2019). Ithemal: accurate, portable and fast basic block throughput estimation using deep neural networks. PMLR, 4505–4515.

[B41] Namolaru M. Cohen A. Fursin G. Zaks A. Freund A. (2010). “Practical aggregation of semantical program properties for machine learning based optimization,” in Proceedings of the 2010 CASES, 197–206.

[B42] Ogilvie W. F. Petoumenos P. Wang Z. Leather H. (2017). “Minimizing the cost of iterative compilation with active learning,” in 2017 CGO, Austin, TX, USA, 04-08 February 2017 (IEEE), 245–256.

[B43] Park E. Cavazos J. Alvarez M. A. (2012). “Using graph-based program characterization for predictive modeling,” in Proceedings of the tenth CGO, 196–206.

[B44] Pekhimenko G. Brown A. D. (2011). “Efficient program compilation through machine learning techniques,” in Software automatic tuning (Springer), 335–351.

[B45] Peng H. Mou L. Li G. Liu Y. Zhang L. Jin Z. (2015). “Building program vector representations for deep learning,” in International conference on knowledge science, engineering and management (Springer), 547–553.

[B46] Pérez Cáceres L. Pagnozzi F. Franzin A. Stützle T. (2017). “Automatic configuration of gcc using irace,” in International conference on artificial evolution evolution artificielle (Springer), 202–216.

[B47] Plotnikov D. Melnik D. Vardanyan M. Buchatskiy R. Zhuykov R. Lee J.-H. (2013). Automatic tuning of compiler optimizations and analysis of their impact. Procedia Comput. Sci. 18, 1312–1321. 10.1016/j.procs.2013.05.298

[B48] Pouchet L.-N. (2012). Polybench/c the polyhedral benchmark suite. Available online at: http://web.cs.ucla.edu/pouchet/software/polybench/ (Accessed March 4, 2026).

[B49] Sher G. Martin K. Dechev D. (2014). “Preliminary results for neuroevolutionary optimization phase order generation for static compilation,” in Proceedings of the 11th workshop on optimizations for DSP and embedded systems, 33–40.

[B50] VenkataKeerthy S. Aggarwal R. Jain S. Desarkar M. S. Upadrasta R. Srikant Y. N. (2020). Ir2vec: llvm ir based scalable program embeddings. ACM Trans. Archit. Code Optim. 17 (4), 1–27. 10.1145/3418463

[B51] Wang Z. Ji S. (2020). Second-order pooling for graph neural networks. IEEE Trans. Pattern Analysis Mach. Intell. 10.1109/TPAMI.2020.2999032 32750778

[B52] Wang Z. O’Boyle M. (2018). Machine learning in compiler optimization. Proc. IEEE 106 (11), 1879–1901. 10.1109/jproc.2018.2817118

[B53] Wang F. Shoshitaishvili Y. (2017). “Angr-the next generation of binary analysis,” in 2017 IEEE Cybersecurity Development (SecDev), Cambridge, MA, USA, 24-26 September 2017 (IEEE), 8–9.

[B54] Wang M. Zheng D. Ye Z. Gan Q. Li M. Song X. (2019). Deep graph library: a graph-centric, highly-performant package for graph neural networks. arXiv preprint arXiv:1909.01315.

[B55] Wang C. Liang Y. Liu Z. Zhang T. Philip S. Y. (2021). “Pre-training graph neural network for cross domain recommendation,” in 2021 IEEE Third International Conference on Cognitive Machine Intelligence (CogMI), Atlanta, GA, USA, 13-15 December 2021 (IEEE), 140–145.

[B56] Weiss K. Khoshgoftaar T. M. Wang D. (2016). A survey of transfer learning. J. Big Data 3 (1), 1–40. 10.1186/s40537-016-0043-6

[B57] Werner M. Servadei L. Wille R. Ecker W. (2020). “Automatic compiler optimization on embedded software through k-means clustering,” in 2020 ACM/IEEE 2nd Workshop on Machine Learning for CAD (MLCAD), Reykjavik, Iceland, 16-20 November 2020 (IEEE), 157–162.

[B58] Young T. Hazarika D. Poria S. Cambria E. (2018). Recent trends in deep learning based natural language processing. Ieee Comput. Intell. Magazine 13 (3), 55–75. 10.1109/mci.2018.2840738

[B59] Zhang Z. Liu S. Yang Q. Guo S. (2021). “Semantic understanding of source and binary code based on natural language processing,” in 2021 IEEE 4th Advanced Information Management, Communicates, Electronic and Automation Control Conference (IMCEC), Chongqing, China, 18-20 June 2021, 2010–2016.

[B60] Zhou K. Dong Y. Wang K. Lee W. S. Hooi B. Xu H. (2021). “Understanding and resolving performance degradation in deep graph convolutional networks,” in Proceedings of the 30th ACM international conference on information & knowledge management, 2728–2737.

